# Communication for coordination: gesture kinematics and conventionality affect synchronization success in piano duos

**DOI:** 10.1007/s00426-017-0893-3

**Published:** 2017-07-21

**Authors:** Laura Bishop, Werner Goebl

**Affiliations:** 1 Austrian Research Institute for Artificial Intelligence (OFAI), Freyung 6/6, 1010 Vienna, Austria; 2 Department of Music Acoustics-Wiener Klangstil (IWK), University of Music and Performing Arts Vienna, Anton-von-Webern-Platz 1, 1030 Vienna, Austria

## Abstract

Ensemble musicians often exchange visual cues in the form of body gestures (e.g., rhythmic head nods) to help coordinate piece entrances. These cues must communicate beats clearly, especially if the piece requires interperformer synchronization of the first chord. This study aimed to (1) replicate prior findings suggesting that points of peak acceleration in head gestures communicate beat position and (2) identify the kinematic features of head gestures that encourage successful synchronization. It was expected that increased precision of the alignment between leaders’ head gestures and first note onsets, increased gesture smoothness, magnitude, and prototypicality, and increased leader ensemble/conducting experience would improve gesture synchronizability. Audio/MIDI and motion capture recordings were made of piano duos performing short musical passages under assigned leader/follower conditions. The leader of each trial listened to a particular tempo over headphones, then cued their partner in at the given tempo, without speaking. A subset of motion capture recordings were then presented as point-light videos with corresponding audio to a sample of musicians who tapped in synchrony with the beat. Musicians were found to align their first taps with the period of deceleration following acceleration peaks in leaders’ head gestures, suggesting that acceleration patterns communicate beat position. Musicians’ synchronization with leaders’ first onsets improved as cueing gesture smoothness and magnitude increased and prototypicality decreased. Synchronization was also more successful with more experienced leaders’ gestures. These results might be applied to interactive systems using gesture recognition or reproduction for music-making tasks (e.g., intelligent accompaniment systems).

## Introduction

Interpersonal communication is critical for joint action tasks like playing piano duets, playing team sports, or dancing, which require collaborators to align their intentions and coordinate their actions in time. Communication during these tasks is continuous and interactive, with collaborators constantly adapting their own intentions and actions in response to the signals they receive from each other (Schiavio & Høffding, 2015). The signals exchanged are typically multimodal (e.g., auditory and visual) and multilayered (e.g., involving facial expressions and body movements simultaneously), and can be subtle, comprising only a raised eyebrow or a brief moment of eye contact (Davidson, 2012). Given these complexities, it is often difficult to determine from a research standpoint exactly what is being communicated and how group members are assimilating incoming information.

Many researchers have used group music-making paradigms to investigate the communication processes underlying interpersonal coordination. Group music-making presents an intriguing context in which to study communication and coordination, since precise coordination must be achieved under inherently ambiguous temporal conditions (even for notated music, timing is only loosely defined by the score). Moreover, the possible means of communication are constrained by the task of performing an instrument, which can limit freedom of movement for much of the body, as well as conventions of public performance, which may prohibit, for example, counting out loud or using a metronome. In recent years, researchers have been applying their knowledge of the communication processes involved in group music-making to computer systems that replicate or react to performer movements (Dahl, 2014; Hoffman & Weinberg, 2011). Such an application requires a detailed understanding of gesture kinematics and how they relate to performers’ intentions.

During skilled ensemble performance, most communication through audio and visual channels is nonverbal. Usually, perception of jointly-produced sound gives sufficient information for performers to coordinate, but visual communication can be important too (Bishop & Goebl, 2015). Visual communication is only rarely a matter of one performer giving directions to another; rather, even if there is a designated leader, collaborating musicians’ body movements interrelate (Chang, Livingstone, Bosnyak, & Trainor, 2017; Moran, Hadley, Bader, & Keller, 2015) and can be mutually influential (Badino, D’Ausilio, Glowinski, Camurri, & Fadiga, 2014). Research has shown that musicians move more predictably when performing with others than when performing alone (Glowinski et al., 2013), a finding that parallels observations made elsewhere in the joint action literature (Hart, Noy, Feniger-Schaal, Mayo, & Alon, 2014; Vesper, van der Wel, Knoblich, & Sebanz, 2011). Visual communication is particularly important in more ambiguous temporal contexts (e.g., at abrupt tempo changes or following long pauses), when co-performers’ interpretations are less certain to align (Bishop & Goebl, 2015; Kawase, 2013).

The current study investigates the gestures that ensemble musicians use to coordinate piece entrances. Typically, at piece entrances, in the absence of a conductor, one musician in an ensemble acts as the leader and gives the others an audio-visual signal to begin. This visual signal should indicate the timing of the first beat as well as the starting tempo for the piece. Ensemble musicians coordinate piece entrances with varying degrees of success. While professionals typically synchronize their first notes with near-perfect precision (at least in concert), students may require several attempts to begin. Synchronization success can vary depending on a range of factors, including musicians’ expertise and familiarity with each other’s playing style, the genre and tempo of the music, the number and combination of instruments, and the presence or absence of a conductor. The aim of our study was to identify factors that contribute to successful coordination at piece onset during piano duo performance. Specifically, we examined how cue gesture kinematics relate to note synchronization.

### Kinematics of effective cueing gestures

Successful coordination is partly dependent on the quality of the visual signal given by the leader—particularly at piece onset, where there is no prior audio to indicate when the first notes should fall or at what tempo. Musicians commonly use head gestures to signal piece onsets, regardless of their instrument; head gestures were therefore our focus here, though we acknowledge that much of the upper body, as well as facial expressions and breathing, can be involved. The current study investigated how head movement kinematics communicate beats, and tested four kinematic properties of head gestures that we predicted could help observers detect communicated beats more successfully. This section of the paper develops these hypotheses.

For both conductors and instrumentalists, the kinematics rather than trajectories of cueing gestures have been shown to indicate the position of the beat, or tactus (Luck & Toiviainen, 2006). “Followers” attempting to synchronize with instrumentalists’ cueing gestures tend to perceive beats as aligning with major peaks in the leader’s head acceleration, rather than with points of direction change in head trajectories (Bishop & Goebl, 2017). This was observed among pianists and violinists in a study of synchronization in duo performance. Performers’ head movements were measured using accelerometers and Kinect sensors as they took turns cueing each other in at the starts of short passages. An aim of the current study was to replicate these findings (H1) using an expanded version of the same procedure and a more precise motion measurement system.

The easiest gestures to synchronize with are presumably those that convey beat position clearly and reliably. If followers aim to align their starting notes with peaks in leaders’ head acceleration, then leader/follower synchronization should be most successful when the leader’s starting notes align precisely with his or her own head acceleration peaks. The current study tested this hypothesis (H2) by calculating the offset of leaders’ first note onsets from major peaks in leaders’ head acceleration curves, and relating the magnitude of these offsets to success in note synchronization.

The clarity of a gesture, and how readily others synchronize with it, might also be influenced by its articulation. Wöllner, Parkinson, Deconinck, Hove and keller, (2012) found that observers synchronized finger-taps more successfully with quantitatively averaged conductor gestures, which were low in jerk, than with individual conductor gestures, which were higher in jerk. Jerk, the third derivative of position, indicates the smoothness of acceleration changes. The authors also observed more successful synchronization with marcato gestures, where the differences between acceleration maxima and minima were large, than with legato gestures, where the differences between acceleration maxima and minima were small. Here, we hypothesized that gestures high in smoothness (low in jerk) would be clearer and synchronized with more successfully than gestures with high jerk (H3).

We also tested the possibility that musicians synchronize more successfully with gestures marked by a larger-magnitude indication of the beat than with gestures marked by a smaller-magnitude indication of the beat (H4). Gesture magnitude was quantified in terms of how far the head travelled along the forwards–backwards axis while indicating the beat. Instrumentalists sometimes exaggerate their gestures at piece entrances and other places where exchanging visual cues benefits synchronization, and a test of how gesture magnitude affects synchronization should indicate whether this is an effective strategy.

Observers’ success at synchronizing with instrumentalists’ cueing gestures might also relate to the prototypicality of those gestures (Wöllner, Parkinson, Deconinck, Hove, & keller, 2012). People synchronize most successfully with gestures that are similar to those they produce themselves (Keller, Knoblich, & Repp, 2007; Wöllner & Cañal-Bruland, 2010). This effect is generally attributed to the strengthening of action prediction mechanisms that occurs with experience. According to this theoretical perspective, people use their own action planning systems to simulate observed movements—a process that may or may not yield overt motor output (Calvo-Merino, Grezes, Glaser, Passingham, & Harrad, 2006; van der Wel, Sebanz, & Knoblich, 2013). They then predict the course of observed gestures using the same mechanisms that they use to predict the course of their own gestures. When the observed or performed gesture is similar to gestures a person has performed in the past, action-perception links are stronger and prediction is facilitated. In the current study, we expected that highly prototypical cueing gestures would be more likely than highly idiosyncratic cues to align with followers’ own gesture tendencies, and would therefore be easier to predict and synchronize with (H5).

### Gesture mimicry to facilitate synchronization

Theories of embodied music cognition posit that we use our own bodies to interpret the musical gestures produced by others (Leman, 2012). In other words, we understand others’ motor intentions by overtly or internally mirroring aspects of their actions (Jacob & Jeannerod, 2005; Jeannerod, 2003). Our ability to internally simulate others’ gestures is thus central to the concept of embodiment. Simulation mechanisms facilitate the translation of gestures into sound and the translation of sound into expressive gestures (Leman & Maes, 2014).

As stated above, observed actions can be simulated without overt replication, though in some cases the process clearly shapes motor output. For example, an imitation bias is observed among people who are asked to make speeded movements that are either congruent or incongruent (e.g., in terms of magnitude or direction) to irrelevant movements viewed simultaneously on a computer screen (Grosjean, Zwickel, & Prinz, 2009; Schubö, Aschersleben, & Prinz, 2001). Incongruent movements are performed less accurately than congruent movements, indicating an unintentional assimilation of observed motion parameters into the observer’s own performed actions.

At times, people overtly mimic each other’s behaviour. This mimicry has cognitive benefits: when people perform gestures that are high in similarity and coordinated in time, their attention is drawn towards each other and their perception and memory for each other’s behaviour is facilitated (Macrae, Duffy, Miles, & Lawrence, 2008). Furthermore, moving in rhythmic coordination with others can promote social bonding, improving participants’ ratings of partner trust and likeability (Hove & Risen, 2009) and increasing the likelihood of prosocial behaviour (Wiltermuth & Heath, 2009).

The current study included a test of whether coordination of body gestures occurs between duo performers at piece onset. Previously, some correlation in head and upper body sway has been observed within pairs of duo pianists. Goebl and Palmer (2009) found that duo pianists’ head movements were more synchronized when they performed under reduced auditory feedback conditions (unable to hear themselves or unable to hear their partner) than when they performed with normal two-way auditory feedback. Despite the heightened synchrony of head movements, however, note synchronization under reduced auditory feedback conditions was poor. Keller and Appel (2010) tracked the upper body movements of piano duos and found that the further the body movements of the primo performer (who usually plays the higher-pitched part) lagged behind those of the secondo (who usually plays the lower-pitched part), the greater note asynchrony became. Since primo note onsets consistently led secondo note onsets, the authors suggested that congruence between leader/follower relations at the levels of note onsets and body sway may be important for successful ensemble coordination.

Still unclear is whether leader/follower coordination of cueing gestures occurs at piece onset, and to what extent this coordination of body movement relates to note synchronization. In line with theories of embodiment, we hypothesized that coordinating cueing gestures would help performers gauge each other’s intended timing and, therefore, facilitate note synchronization (H6). Followers’ gestures were expected to mimic the form of leaders’ gestures and follow a parallel timecourse.

### Current study

This study aimed to assess how kinematic measures affect the synchronizability of ensemble musicians’ cueing-in gestures. Motion capture recordings were made of nine piano duos performing short musical passages under alternating leader and follower conditions. The assigned leader for each trial was responsible for cueing in the follower at a particular tempo, with the aim of synchronizing their performance of the passage as precisely as possible. During a subsequent gesture-following task, a subset of the motion capture recordings of leader performances were presented (with audio) to an independent sample of 10 skilled musicians, who tapped in time with the leaders’ performed beats. Using data from this test, a measure of “synchronizability” (i.e., average leader–follower first beat asynchrony) was obtained for each leader gesture.

The alignment between followers’ first taps (for gesture-following task participants) or performed beats (for interactive duo performance task participants) and extremes in leaders’ head position, velocity, and acceleration curves was examined. Our focus was exclusively on leader–follower synchronization at piece onset, though a similar investigation of how gesture kinematics affect synchronization across the first few beats of a piece could also be made. It was expected that followers’ first taps would align with acceleration peaks in leaders’ gestures (H1), confirming previous findings (Bishop & Goebl, 2017). It was also expected that the precision of alignment between leaders’ first note onsets and their own head acceleration peaks (H2), as well as increased gesture smoothness (H3), magnitude (H4), and prototypicality (H5) would improve the synchronizability of leaders’ gestures. Finally, the hypothesis that increased similarity in the movements made by leader–follower pairs at the time of piece onset relates to improved leader–follower note synchronization was tested (H6), using data from the recording sessions.

## Methods

### Interactive duo performance experiment

Our first experiment investigated pianists’ synchronization with their duo partners’ cueing-in gestures under interactive conditions, while two-way communication was possible. The hypothesis that followers synchronize their piece onsets with peaks in leaders’ head acceleration was assessed. We also tested for coordination in duos’ body movements around piece onsets.

#### Participants

Eighteen pianists (10 female) recruited from among the students at the University of Music and Performing Arts Vienna completed the experiment. Our sample size was set with the aim of obtaining enough recorded performances to carry out the gesture-following task. Six pianists had minimal experience playing the piano in small ensembles, six had extensive experience, and six were completing a degree in either choral or orchestral conducting. Some pianists (10 of the 18) had completed a similar version of the task for the experiment reported in Bishop and Goebl (2017). Some of the pianists knew their assigned partner, but none had performed together before. Participants provided informed consent before completing the experiment and received a small travel reimbursement.

“Conductor”, “ensemble-experienced”, and “ensemble-inexperienced” groups did not differ significantly in terms of age (conductors *M* = 28.0, SD = 7.5; ensemble-experienced *M* = 27.2, SD = 4.7; ensemble-inexperienced *M* = 25.2, SD = 3.4; *F*(1, 13) = 0.37, *p* = 0.55) or years of piano-playing experience (conductors *M* = 17.0, SD = 7.4; ensemble-experienced *M* = 22.0, SD = 5.6; ensemble-inexperienced *M* = 17.8, SD = 3.4; *F*(1, 13) = 1.64, *p* = 0.22). However, the ensemble-experienced group had more experience playing in duos and other small ensembles (self-rated *M* = 12.7 out of 15, SD = 1.4; conductors *M* = 8.5, SD = 2.1; ensemble-inexperienced *M* = 8.2, SD = 1.3; *F*(1, 13) = 18.45, *p* = 0.001, $\eta ^2=0.59$). Only the conductors had prior conducting experience (*M* = 3 years, SD = 1.7).

**Table 1:** Musical stimuli are listed with their starting tempi and meters

Composer	Piece	Tempo	Meter
André	Sonata Facile, Op. 56	100	4/4
J.C.F. Bach	Sonata in C major for 4 Hands	90	2/2
Beethoven	String Quartet No. 3, Op. 18	65	2/4
Diabelli	Jugendfreude, Op. 163, No. 2	220	4/4
Diabelli	Sonates Mignonnes, Op. 150, No. 1	70	4/4
Haydn	Divertimento in G major	95	2/4
Haydn	String Quartet in G major, Op. 76	65	2/4
Kuhlau	Rondo, Op. 111	135	2/4
Löschhorn	Kinderstücke, Op. 182, No. 6	45	2/2
Mozart	Divertimento in F major, KV. 138	135	4/4
Mozart	Piano Sonata in B-flat major, K. 358	160	4/4
Pleyel	Quartet in A, Op. 20, No. 2	120	4/4
Ravel	Quartet in F major	110	4/4
Schubert	Overture in F major for 4 Hands	60	2/2
Strauss	Sperl-Polka, Op. 133	135	2/4

Tempo values are per half note for passages with a a 2/2 m, and per quarter note otherwise

#### Stimuli and equipment

**Fig. 1:**
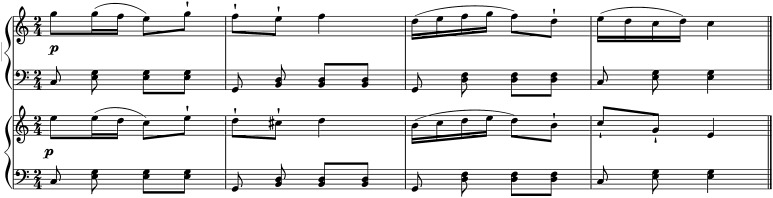
Sample piano duet stimulus: primo (*upper*) and secondo (*lower*) parts for the passage based on Kuhlau’s Rondo, Op. 111

Pianists performed 15 passages adapted from the starts of pieces in the Western classical repertoire (Table 1). Some further details on these pieces are given in Bishop and Goebl (2017). A sample piece is shown in Fig. 1. All passages were in duple meter, 2–4 bars in length, multi-voiced (to be played with both hands), and adapted so that the two performers would always start in unison on the first downbeat. Pieces that were likely to be unfamiliar to participants were chosen to encourage communication between duo members and to ensure that they would not have preexisting expectations regarding the tempo. A tempo was selected for each passage based on the original tempo indications in the score; these ranged from 45 to 220 bpm, with approximate mean interbeat intervals of 111 ms at the slowest tempo and 1000 ms at the fastest tempo.

**Fig. 2:**
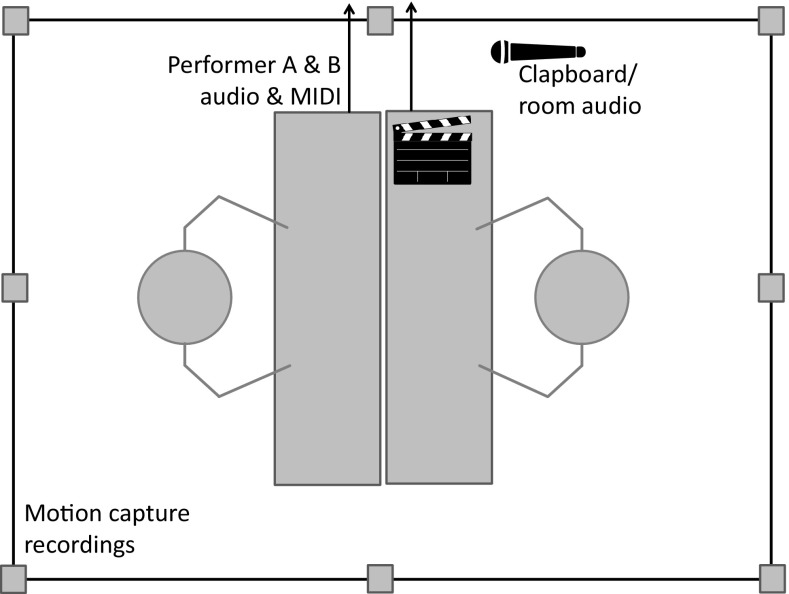
Illustration of recording set-up

Pianists performed on two Yamaha CLP470 Clavinovas and faced each other directly, as shown in Fig. 2. Audio and MIDI from the Clavinovas were recorded via a Focusrite Scarlett 18i8 sound card in Ableton Live, along with audio from a standing microphone placed between the two performers (44.1 kHz sampling rate).

Pianists’ upper body movements were recorded using an eight-camera (Prime 13) OptiTrack motion capture system. Each pianist wore a jacket and cap, to which 25 spherical markers were affixed (including three on the head). Marker positions were recorded at a rate of 240 frames per second.

To synchronize audio/MIDI and motion capture data, a film clapboard was placed on top of one of the Clavinovas with an OptiTrack marker attached and struck once at the start and end of each block. These claps were clearly discernible in the OptiTrack data and in the audio recorded by the standing microphone, which was recorded in synchrony with the audio and MIDI from the Clavinovas.

#### Procedure

Pianists were given hard copies of the passage scores at the start of the recording session and had time to practice together. The recording phase began once both performers could play the passages without error.

Recording sessions were divided into two blocks. In each block, the performers played once through each of the 15 passages (in a pseudorandomized order, structured so that passages with a similar tempo were played consecutively). Each performer was instructed to play either the part labelled “A” or the part labelled “B”; these indicated primo and secondo lines and were assigned pseudorandomly, so that each performer played about the same number of primo and secondo parts (7 or 8 of each). Thus, each participant played a total of 30 trials, going once through the 15 passages in each block.

Leader/follower roles were assigned on an alternating basis, so each performer led each passage once. At the start of each trial, the assigned leader was handed a pair of headphones and listened to a metronome beat indicating the tempo for the passage. They then returned the headphones to the experimenter before beginning to play. The leader’s task was to coordinate the entrance of the passage without speaking (e.g., counting out loud). Duos were instructed to focus on playing together and to ignore pitch errors as much as possible, but if major timing or pitch errors made it impossible to continue, they were allowed to redo the trial. Once the recordings were finished, pianists completed a musical background questionnaire.

### Analysis

#### Alignment of audio/MIDI and motion capture data

The experimenter struck a film clapboard at the start and end of each block (see “Stimuli and equipment”). The initial strike was used as a reference “time 0”, and the timestamps for all recordings were recoded to indicate elapsed time since this point. To check the precision of this inter-recording alignment, for each recording, the interval between first and second clapboard strikes was calculated, and discrepancies between recording devices in interval lengths were assessed. The mean discrepancy was minimal, less than the duration of one sample of motion capture data (*M* = 2.9 ms, SD = 2.4).

#### Gesture position, velocity, acceleration, and jerk

Motion capture data comprised series of *x*, *y*, and *z* axis coordinates for 25 upper body markers, indicating forwards/backwards, left-right, and up/down movement, respectively. Here, we report only on the motion of the front-most head sensor (positioned slightly above the forehead), as motion was also measured from this location in Bishop and Goebl (2017), and the current study was partially designed to validate our earlier findings. For analyses of position and velocity, only forwards−backwards (*x* axis) data were used. For analyses of acceleration, a 3D measure was computed using the square root of the sum of squares for *x*, *y*, and *z* axes, with gravity added into the *y* dimension (gravity was included, again, for the purpose of equating our measures with the earlier work).

Gesture position data were smoothed using functional data analysis (Ramsay & Silverman, 2002; Goebl & Palmer, 2008). Order-7 b-splines were fit to the trajectories with knots every 50 ms, applying a roughness penalty on the fifth derivative ($\lambda = 10^{-18}$), which smoothed the third derivative (jerk). The functional data were then converted back for further analysis with samples every 5 ms.

Motion data were segmented into trials, based on visual analysis of the motion capture recordings. A “cue window” was then identified in each trial, comprising the two interbeat intervals prior to the leader’s first note onset and the leader’s first performed interbeat interval. Interbeat intervals were defined as the duration of a quarter note for pieces written in 4/4 and as the duration of a half note for pieces written in 2/2. Any cueing-in gestures that were given would fall within that window.

#### Primo-secondo note asynchronies

MIDI data from the Clavinovas were aligned with the corresponding notation using the performance-score matching system developed by Flossmann, Goebl, Grachten, Niedermayer and Widmer (2010), which pairs MIDI pitches with score notes according to pitch sequence information. Only pitch sequence is considered, so rhythm errors are not penalized. Mismatched pitches resulting from performer error or incorrect interpretation of the pitch sequence by the matching system can be corrected via a graphical user interface. Matched performances thereby include only correctly performed and correctly matched notes. The mean pitch error rate across all completed performances (i.e., excluding false starts, but including all other notes) was 9.5% (SD = 8.8%). Using these matched performances, primo-secondo asynchronies were calculated for notes that should have been synchronized, according to the score. Asynchronies were calculated for the entirety of each performance, but for the analyses presented here, we used the asynchronies achieved on the first chord of each piece as our main dependent variable. Asynchronies were not normally distributed, so non-parametric tests were used.

### Gesture-following experiment

A second experiment was carried out with the aim of identifying the kinematic properties that improve cueing gesture synchronizability. Audio-visual recordings of pianist performances, collected during the first experiment, were used as stimuli for a beat-tapping task, which was completed by a sample of 10 musicians. The average accuracy of these musicians’ synchronization was taken as an indicator of gesture synchronizability, serving as a more reliable measure than the accuracy of individual followers’ synchronization during the interactive duo performance experiment, due to the larger sample size.

#### Participants

Ten skilled musicians (2 female) completed the task (age *M* = 28.1, SD = 3.5). Their experience covered a variety of instruments, including piano (5 musicians), violin (2), saxophone (1), voice (1), and percussion (1). They reported an average 19.8 years of instrument-playing experience (SD = 2.8), and gave an average self-rating of 11.2 out of 15 (SD = 1.8) to describe the extent of their experience playing in duos and other small ensembles. All participants provided written informed consent.

#### Stimuli and equipment

**Fig. 3:**
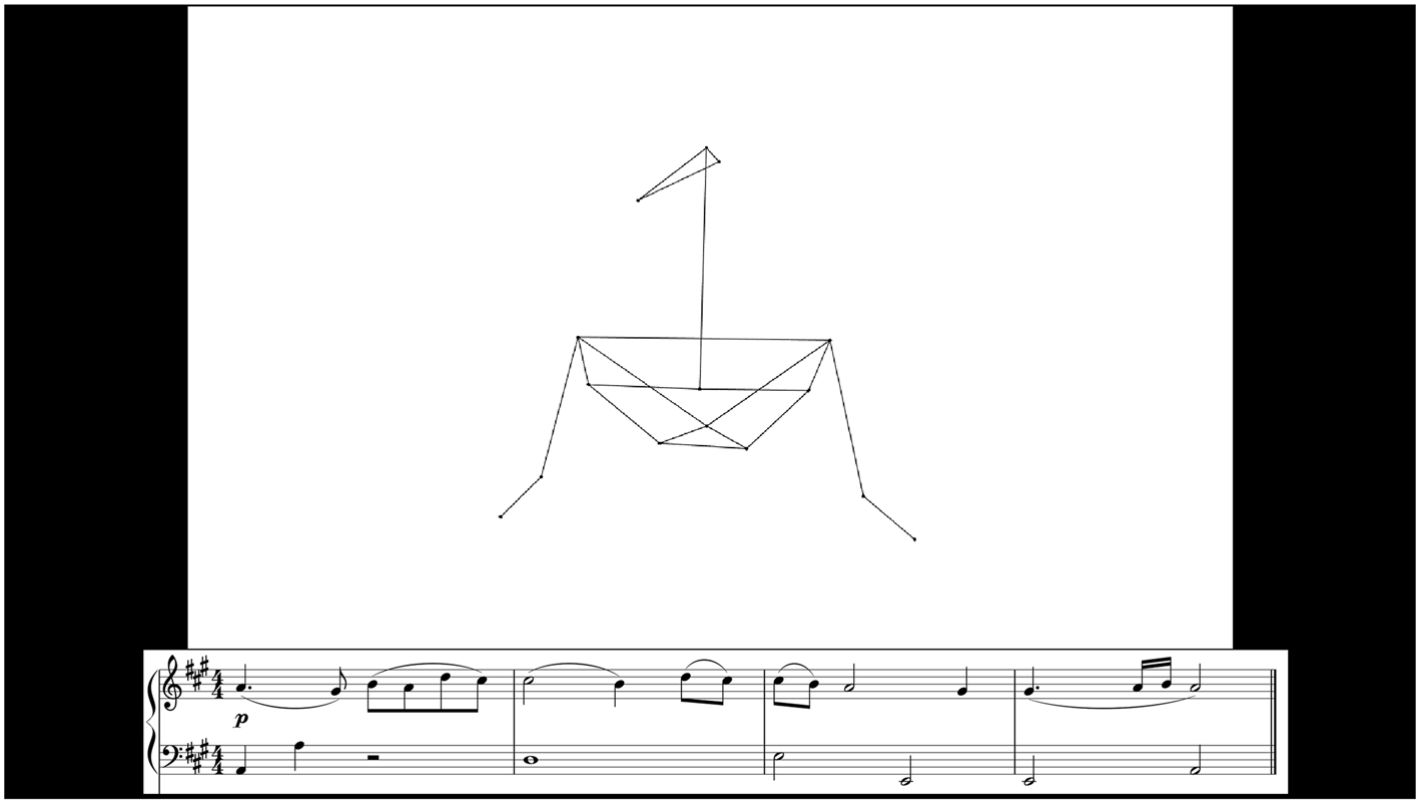
Sample display for an experimental trial during the cue quality test

Musicians were presented with audio–video clips of 108 (of the total 270) leader performances recorded during the main experiment. Only leader performances were used, since they contained the cueing-in gestures. Twelve of the performances with the highest note accuracy were selected at random from each duo. The selected performances represented a wide range of tempi (36–210 bpm; median 82.6 bpm)—nearly the full range used in the interactive duo performance task (listed in Table 1). The videos featured a point-light representation of the leader, shown as black dots connected by straight black lines on a white background (Fig. 3). Point-light figures excluded hand markers and hip markers, as these had not been visible to followers during the original performances, but included the head (front, top, and right markers) and all other upper body markers.

3D images of each frame were drawn up in R (using the “rgl” package), then combined into videos in VideoMach. Presentation (Neurobehavioral Systems), running on an HP EliteBook, was used to display videos and play the accompanying temporally-aligned audio.^1^ Only leaders’ audio was presented. Videos were displayed in a 1280 × 720 pixel box on a black screen and played at 60 fps. The corresponding musical notation was shown below the video display, in a 1200 × 180 pixel box. During familarization trials (see below), the complete score for each passage (including both primo and secondo parts) was presented in a 1250 × 500 pixel box, and the full (primo and secondo) audio recordings were played.

Participants’ tapping responses were made on the middle C key of one of the Clavinovas used during the recording sessions. (A Clavinova was used so that the motor requirements would be similar to those encountered by the pianists who made the recordings.) The output volume was turned off, so participants heard only the thud of their finger on the key in addition to the stimulus audio. The PC presenting stimulus clips was placed on top of one of the Clavinovas. So that MIDI response data could be aligned with video timestamps, a photoresistor was taped to the top of the computer screen, at the corner of the video display. The photoresistor registered the change in lighting that occurred the start of each trial; this information was transmitted as audio data and recorded via a Focusrite sound card in Ableton, in sync with the MIDI data from the Clavinova.

#### Procedure

The experiment was completed in six blocks. The first block constituted a familiarization phase: scores for 8 of the 15 passages were presented along with the corresponding audio tracks (one duo’s recording of each passage was selected at random from those with good synchronization and few pitch errors). Participants were instructed to tap along with the beat of each sounded passage, aligning their first taps as closely as possible with the first notes. The purpose of this block was twofold: (1) to familiarize participants with the passages they were about to hear and (2) to obtain a measure of the delay between piece onsets and first taps when participants could only react to the start of the music, not predict it. Participants were free to choose at which hierarchical level of the beat they tapped (e.g., per eighth note, quarter note, or half note, depending on the tempo of the piece).

Two blocks of 25 experimental trials were then completed, starting with five practice trials. Practice trials were supervised by the experimenter, who reminded participants of the task instructions as necessary. At the start of each trial, the score for the upcoming performance was presented; participants were free to look it over, then pressed a key on the computer keyboard to start the audio–video recording. They were again instructed to tap along with the beat of the music, using the video recordings to help in aligning their first taps with piece onsets. Only recordings of the pieces presented during the first familiarization phase were included in these blocks.

The second half of the experiment followed the same pattern as the first. A second familiarization block was completed, in which scores for the remaining seven passages were presented and participants tapped along with the beat of the corresponding audio. Two blocks of 29 experimental trials followed. At the end of the session, participants completed a musical background questionnaire.

**Fig. 4:**
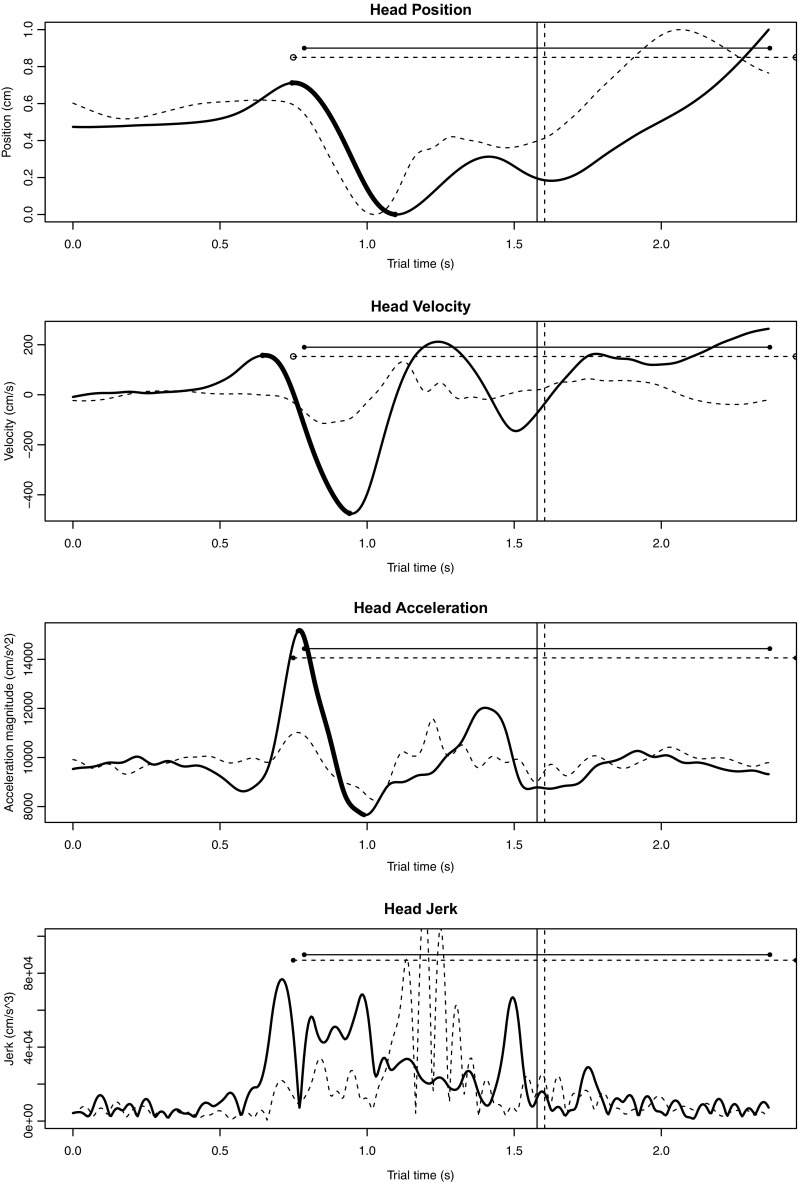
Head position, velocity, acceleration, and jerk curves for one performance of a duo in the ensemble-inexperienced group. Each plot shows the pianists’ movements during the cue window. The *solid lines* indicate the leader’s movements and the *dotted lines* indicate the follower’s movements. *Vertical lines* have been added to show the position of the performers’ first note onsets; *horizontal lines* show the length of their first IBIs. The *bolded* segments of the leader’s position, velocity, and acceleration *curves* indicate the peak-trough pair that was identified as corresponding to the main cue gesture

## Results

### Indicators of beat position

A central aim of this study was to test the hypothesis that points of peak acceleration in instrumentalists’ cueing gestures communicate beat position (H1). This prediction was addressed with an analysis of how followers’ first note onsets aligned with leaders’ gestures. More specifically, we assessed the alignment between followers’ first onsets and extremes in leaders’ head position, velocity, and acceleration curves. If beats were to be communicated via head position, it is logical to expect that beat locations would align with points of path reversal, as these occur in all gestures, regardless of their trajectory. If beats were to be communicated via head velocity or acceleration, they would likely coincide with either maxima or minima in the velocity or acceleration curves.

Peaks and troughs, therefore, were identified in the cue window of each leader’s position, velocity, and acceleration curves. Peaks were defined as points preceded by five consecutively increasing observations and followed by five consecutively decreasing observations that were outside the 99% confidence interval for a surrounding window of 300 ms. Troughs were defined as points preceded by consecutively decreasing observations and followed by five consecutively increasing observations that were likewise outside the 99% confidence interval for a surrounding 300 ms window. A constant rather than tempo-adjusted window size was used, as adjusting for tempo would have required a subjective judgement of at what hierarchical level of the beat each performer had gestured (e.g., 2 beats per bar vs. 4 beats per bar).

Cue gestures were assumed to have at least two points of path reversal, so peak-trough pairs separated by no more than one beat were identified. Since we expected the cue gesture to be more prominent than other movements made during the cue window, the peak-trough pair spanning the greatest range in position, velocity, or acceleration values was selected. The time interval between each selected peak and trough and the follower’s first note onset was calculated as an indication of the precision of their alignment. Peak-to-onset and trough-to-onset intervals were averaged across trials to produce a mean interval for each follower. Interval durations were divided by performers’ average interbeat intervals to achieve normalized values with units of interbeat intervals (IBIs). Sample head position, velocity, acceleration, and jerk curves are given in Fig. 4.

**Table 2:** Peak-to-onset and trough-to-onset distribution medians and standard deviations (in IBIs) for followers from both experiments

Motion parameter	Point	Interactive duo performance task			Gesture-following task		
		Aligned (%)	Median	SD	Aligned (%)	Median	SD
Head position	Peak	44	0.90	0.25	42	1.05	0.45
	Trough	56	0.22	0.31	34	0.43	0.44
Head velocity	Peak	22	0.80	0.27	38	1.10	0.42
	Trough	39	0.28	0.36	38	0.56	0.44
Head acceleration	Peak	50	0.89	0.24	50	1.09	0.28
	Trough	33	0.71	0.28	63	0.89	0.29

These values indicate the alignment between leaders’ gestures and followers’ first performed beats during the cue window; positive medians indicate that leaders’ gestures preceded followers’ beats. The percentages of followers whose peak- or trough-to-onset intervals aligned approximately with 0 or 1 IBI are also listed

If either peaks or troughs in a given dimension indicate beats, then peak- or trough-to-onset intervals could be expected to cluster around two points: interval lengths of approximately 1 IBI would occur if the selected gesture feature preceded the follower’s onsets by one IBI (communicating a preparatory beat), while interval lengths of approximately 0 IBIs would occur if the feature and the follower’s onsets were synchronized. For our purposes, a clustering of intervals around either value was taken as an indication that the point communicated beat position. To assess the reliability of alignment between the selected peaks and troughs and followers’ first onsets, the proportion of average intervals approximating either 0 IBIs or 1 IBI (±0.2 IBIs) was calculated. Separate analyses were done for followers from the interactive duo performance task and gesture-following task.

Statistics for peak- and trough-to-onset interval distributions are presented in Table 2. To make sense of these data, we have to consider both (1) how reliably followers’ onsets aligned with each landmark and (2) around which values each distribution centered. Reliable alignment with a particular landmark (high percentages in columns 3 and 6 of Table 2), plus a median value near 0 or 1 IBI, would be evidence that the landmark communicates beat position.

**Fig. 5:**
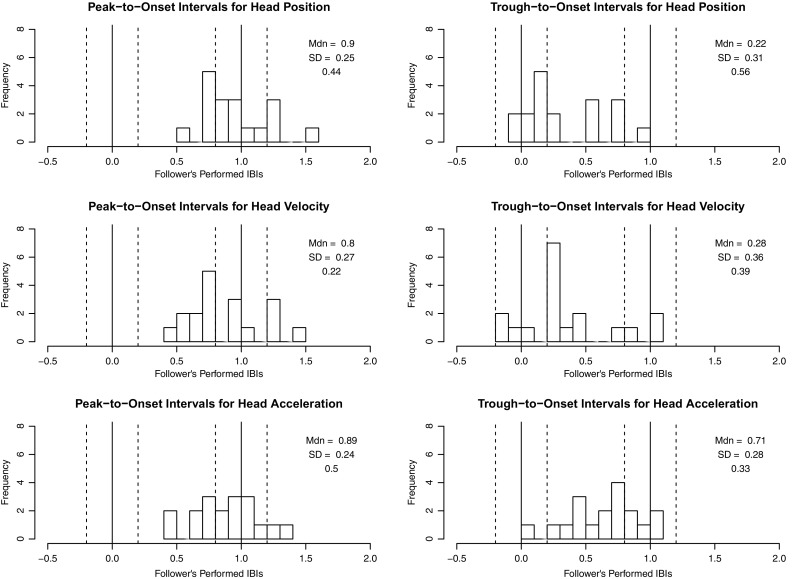
Distributions of peak-to-onset and trough-to-onset intervals for followers in the interactive duo performance task. Intervals within the *vertical dotted lines* were counted as approximately equivalent to 0 or 1 IBI in length. The proportion of intervals that fell within these ranges are given for each distribution, along with the distribution median and standard deviation

**Fig. 6:**
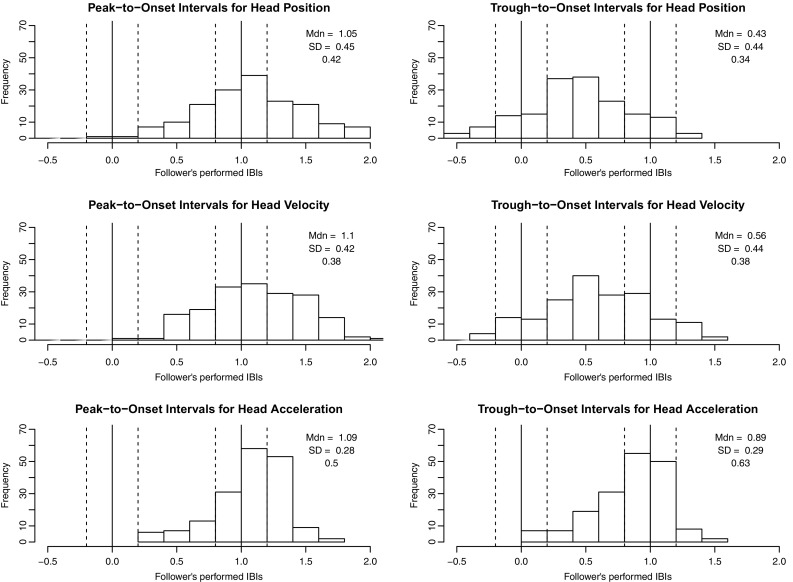
Distributions of peak-to-onset and trough-to-onset intervals for gesture-following task participants. Intervals within the *vertical dotted lines* were counted as approximately equivalent to 0 or 1 IBI in length. The proportion of intervals that fell within these ranges are given for each distribution, along with the distribution median and standard deviation

Interval distributions for the interactive duo performance and gesture-following tasks are shown in Figs. 5 and 6, respectively. For head position, we found that followers in both tasks aligned their first onsets more closely with peaks than troughs. Medians for peak-to-onset distributions were near 1 IBI, while medians for trough-to-onset distributions were not. For head velocity, neither peaks nor troughs seemed to communicate beats, as alignment percentages were low and medians were not reliably near to either 0 or 1 IBI. For head acceleration, the results were a bit more complex: followers aligned their onsets more closely with peaks than troughs in the interactive task, while in the gesture-following task, onsets aligned slightly more closely with troughs than peaks. The period of deceleration between acceleration peak-trough pairs might have communicated beats to participants in the gesture-following task—a possibility that is considered in the discussion. For both tasks, however, followers aligned their first onsets more reliably with acceleration landmarks than with position or velocity landmarks.

Across motion parameters, a timing difference was noticeable between the interactive duo performance and gesture-following tasks: gesture-following participants’ first taps tended to align with a later point on leaders’ gesture curves than did interactive duo followers’ first onsets. For example, peaks in position and acceleration preceded interactive duo followers’ first onsets by slightly less than one beat and gesture-following participants’ first taps by slightly more than one beat. Correspondingly, leader-follower asynchronies were greater for gesture-following participants than for interactive duo participants, *Z* = 20.44, *p* < 0.001, *r* = 0.56 (gesture-following task *M* = −0.16 IBIs, SD = 0.64 IBIs; interactive duo task *M* = −0.01 IBIs, SD = 0.17).^2^ This timing difference could reflect better anticipation of the beat among interactive duo followers or a task-dependent difference in how beats were perceived.

The potential effects of leader gesture/follower onset alignment on note synchronization were assessed as an additional test of which gesture parameters were most useful in communicating beat position during the gesture-following task. Only position peak-to-onset, velocity peak-to-onset, and acceleration peak- and trough-to-onset interval distributions were considered, since their medians were close to 1 IBI (Table 2, column 7). For each distribution, trials with intervals approximating 0 or 1 IBI (±0.1 IBIs; “aligned”) were compared to trials without intervals approximating 0 or 1 IBI (“not aligned”), using mean absolute asynchronies of first tapped beats as the dependent variable. Significantly improved synchronization (at $\alpha =0.01$) was observed when first taps aligned with position peaks, *Z* = 2.95, *p* = 0.003, *r* = 0.11 (aligned *M* = 0.33 IBIs, SD = 0.29; not aligned *M* = 0.41 IBIs, SD = 0.33), acceleration peaks, *Z* = 4.83, *p* < 0.001, *r* = 0.15 (aligned *M* = 0.33 IBIs, SD = 0.32; not aligned *M* = 0.41 IBIs, SD = 0.34), and acceleration troughs, *Z* = 7.13, *p* < 0.001, *r* = 0.22 (aligned *M* = 0.27 IBIs, SD = 0.27; not aligned *M* = 0.42 IBIs, SD = 0.34). No significant difference was observed for velocity peaks, *Z* = 2.02, *p* = 0.04. These findings provide evidence that leaders’ head trajectories and acceleration patterns are used as cues to beat position.

### Gesture properties that support successful synchronization

In this section, analyses testing the potential effects of gesture kinematics and leader expertise on leader–follower synchronization are presented, using data from the gesture-following task. Asynchronies obtained from the gesture-following task were not normally distributed, so the results of non-parametric tests are reported.

#### Alignment between leaders’ gestures and sounded performance (H2)

Increased precision in the alignment between leaders’ first note onsets and their own cueing gestures was expected to facilitate leader–follower note synchronization. To test this hypothesis, the time intervals between leaders’ first note onsets and peaks and troughs in their head position, velocity, and acceleration curves were assessed, using the same analysis procedure as described in the previous section. This analysis had the additional effect of clarifying which kinematic landmarks correspond to leaders’ intended beats.

Interval distribution statistics are presented in Table 3. As we saw for followers, leaders’ first onsets aligned more closely with peaks than troughs in head position—only the peak-to-onset interval distribution median was close to 1 IBI. Leaders’ first onsets did not reliably align with either velocity peaks or troughs. For acceleration, alignment was more precise and reliable with peaks than troughs, as evidenced by the peak-to-onset distribution median near 1 IBI and the relatively high proportion of leaders whose average peak-to-onset intervals approximated 1 IBI in length. Leaders’ onsets aligned slightly more reliably with acceleration peaks than with position peaks, as we saw in the previous section for followers’ onsets.

We also tested whether note synchronization was more successful in the gesture-following task on trials where leaders’ head position or acceleration peaks either aligned with or preceded their own first onsets by 1 IBI (±0.1 IBIs) than on other trials. The difference in mean absolute note asynchronies was significant (at $\alpha =0.03$) for position peaks, *Z* = 2.44, *p* = 0.01, *r* = 0.08 (aligned *M* = 0.32 IBIs, SD = 0.22; not aligned *M* = 0.40, SD = 0.33), but not acceleration peaks, *Z* = 1.01, *p* = 0.31 (aligned *M* = 0.36, SD = 0.27; not aligned *M* = 0.41, SD = 0.35). The alignment of leaders’ first onsets with peaks in their own head trajectories, therefore, systematically improved note synchronization.

**Table 3:** Peak-to-onset and trough-to-onset distribution means and standard deviations (in IBIs) for leaders

Motion parameter	Point	Leader onsets		
		Aligned (%)	Median	SD
Head position	Peak	50	0.89	0.24
	Trough	50	0.22	0.29
Head velocity	Peak	44	0.83	0.28
	Trough	44	0.31	0.35
Head acceleration	Peak	61	0.90	0.21
	Trough	39	0.71	0.24

These values indicate the alignment between leaders’ gestures and leaders’ first note onsets during the cue window. The percentages of leaders whose intervals approximated 0 or 1 IBI in length are listed under “Aligned”

#### Gesture smoothness and magnitude (H3–4)

Better synchronization was expected with gestures that were smooth than with gestures that were high in jerk. Better synchronization was also expected with gestures that provided a large rather than small magnitude indication of the beat. For each trial, an average value of 3D gesture jerk was calculated (using the root sum squared of jerk values in *x*, *y*, and *z* dimensions), and a measure of gesture magnitude (calculated as the spatial distance between the leader’s maximum and minimum head positions) was obtained. The degree of correlation between these values and the mean absolute asynchronies achieved by participants in the gesture-following task on their first tap of each trial were assessed. There was a positive correlation between mean gesture jerk and mean absolute asynchronies, $\tau =0.22$, *z* = 3.26, *p* = 0.001 (significant at $\alpha =0.03$), suggesting a tendency for asynchrony to increase with increasing jerk. Gesture magnitude correlated slightly but significantly with mean absolute asynchronies, $\tau =-0.19$, *z* = 2.48, *p* = 0.01, indicating that asynchronies decreased as gesture magnitude increased.

#### Gesture prototypicality (H5)

**Fig. 7:**
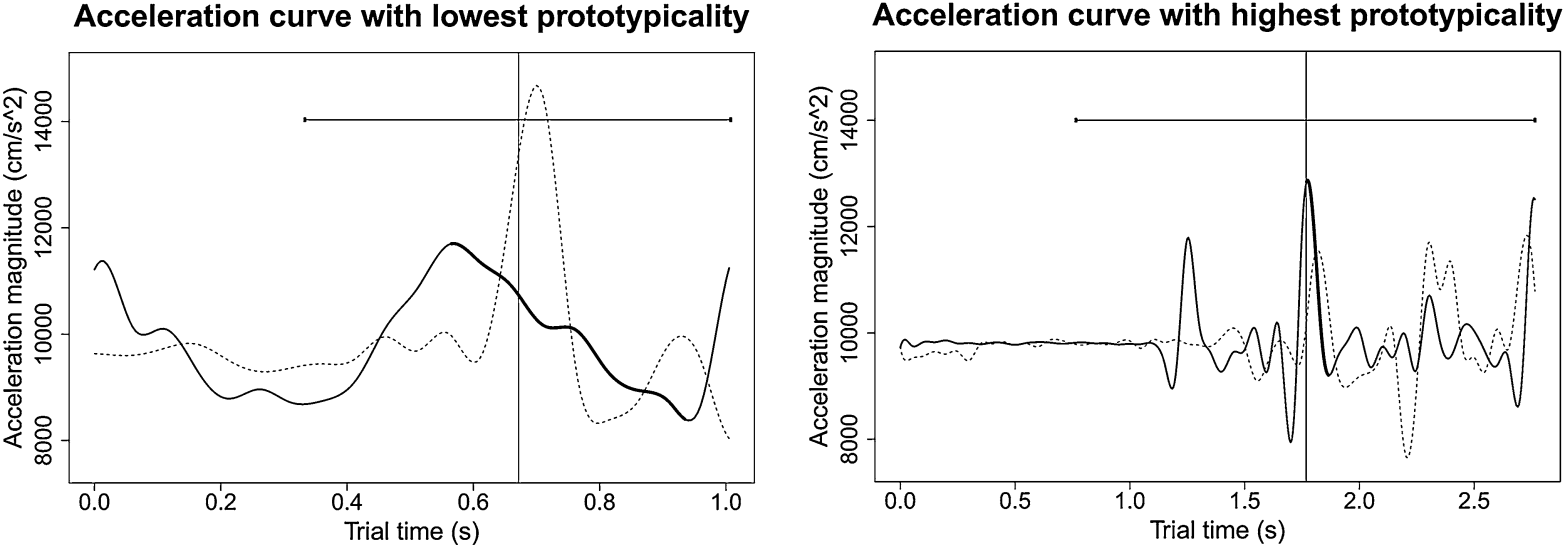
Leader acceleration curves with the lowest (*left*) and highest (*right*) measured prototypicality values. Recording followers’ acceleration curves are shown as *dotted lines*. *Vertical lines* indicate the position of leaders’ first note onsets; *horizontal lines* mirrored on either side of the onset *line* indicate the duration of leaders’ first performed IBI. The curve segment corresponding to the main cue gesture is in *bold*. The mean absolute asynchrony across gesture-following task participants was 0.88 IBIs for the gesture with the lowest prototypicality and 0.35 IBIs for the gesture with the highest prototypicality

Gestures that followed prototypical patterns of motion were expected to encourage more successful synchronization than gestures that followed idiosyncratic patterns of motion. To obtain a measure of “gesture prototypicality”, we evaluated how similar each gesture was to all other gestures in the stimulus set. Cross-correlations were calculated between all recorded leaders’ cue gestures, within and between duos. For each gesture, a mean absolute lag-0 correlation magnitude was then computed. The acceleration curves with the lowest and highest prototypicality (i.e., highest and lowest mean correlation magnitudes, respectively) are shown in Fig. 7.

Correlations were calculated between average lag-0 correlation magnitudes and the mean absolute asynchronies achieved by participants in the gesture-following task, on the first beat of each trial. Positive correlations (at $\alpha =0.02$) were observed for head position, $\tau =0.19$, *z* = 2.87, *p* = 0.004, velocity, $\tau =0.38$, *z* = 5.81, *p* < 0.001, and acceleration, $\tau =0.25$, *z* = 3.89, *p* < 0.001, indicating that as gesture prototypicality increased, mean asynchronies also increased. Thus, contrary to our hypothesis, followers synchronized less successfully with leaders who gave more prototypical gestures.

#### Evaluating predictors of synchronization success

The potential value of the gesture attributes discussed above as predictors of followers’ synchronization success was evaluated via multiple regression. A (non-hierarchical) model was constructed that included (1) leader experience group (ensemble-inexperienced, ensemble-experienced, conductor-pianists), (2) leaders’ note alignment with their own head acceleration peaks, (3) gesture jerk, (4) gesture magnitude, and (5) gesture prototypicality as predictors. Mean absolute asynchronies achieved by participants in the gesture-following task on their first taps served as the dependent variable.

The overall model was significant, *F*(5, 720) = 27.32, *p* < 0.001, (adjusted) $R^2=0.18$. It accounted for a low proportion of variance, but this is not surprising given the number of factors involved in synchronizing with visual cues. Significant effects at an adjusted $\alpha =0.01$ were observed for gesture magnitude, *t*(720) = 3.73, *p* < 0.001, $\eta ^2=0.02$, gesture jerk, *t*(720) = 2.96, *p* = 0.003, $\eta ^2=0.01$, and gesture prototypicality, *t*(720) = 10.24, *p* < 0.001, $\eta ^2=0.12$. We also found a significant effect of leader experience: synchronization was more successful with ensemble-experienced pianists’ gestures than with ensemble-inexperienced pianists’ gestures, *t*(720) = 4.77, *p* < 0.001, $\eta ^2=0.03$, and more successful with conductor-pianists’ gestures than with ensemble-inexperienced pianists’ gestures, *t*(720) = 3.35, *p* < 0.001, $\eta ^2=0.81$. The effect of leader gesture-note alignment, *t*(720) = 1.61, *p* = 0.11, was not significant. We can conclude, therefore, that increased ensemble and conducting experience, increased gesture smoothness and gesture magnitude, and decreased gesture prototypicality contribute to improved follower synchronization.

### Gesture coordination and note synchronization in interactive duo performance task

#### Similarity in leader–follower gesture patterns (H6)

It was hypothesized that, during the interactive duo performance task, some followers would make gestures that were similar in timing and form to the gestures made by leaders. To assess the similarity in movements made by leaders and followers, cross-correlation functions were calculated between leaders’ and followers’ head position, velocity, and acceleration curves, for each trial, in intervals of 15 ms, up to a maximum lag of three IBIs.

For each trial, the lag with the strongest absolute correlation was identified. Positive correlations indicated that the leader and follower were moving in-phase; negative correlations indicated that they were moving in anti-phase. Correlation values and corresponding lags are reported in Table 4. Moderate negative correlations were observed between absolute maximum correlation values and their corresponding lags for position, $\tau =-0.27$, *z* = 6.56, *p* < 0.001, velocity, $\tau =-0.26$, *z* = 6.33, *p* < 0.001, and acceleration curves, $\tau =-0.25$, *z* = 6.28, *p* < 0.001 (all significant at $\alpha =0.02$), suggesting that when leader and follower gestures aligned more closely in time, the degree of similarity in their movements also increased.

**Table 4:** Leader–follower cross-correlations

Motion parameter	Max correlation strength		Max correlation lag (IBIs)	
	Mean	SD	Mean	SD
Position	0.69	0.15	−0.24	0.64
Velocity	0.56	0.22	−0.40	0.59
Acceleration	0.55	0.21	−0.41	0.63

Means and SDs for (left) the strongest correlation values observed in the cross-correlation profiles calculated for each trial and (right) the corresponding lags. Negative mean lags indicate that followers’ gestures lagged behind leaders’ gestures

We also examined whether greater temporal alignment in performed gestures related to note synchronization. As a measure of temporal alignment between gestures, we used the lag that corresponded to the maximum correlation value. When maximum correlations occurred at lags close to 0, this would indicate high temporal alignment between leader and follower. Mean absolute note asynchronies achieved on trials in which maximum correlations occurred close to lag 0 (±0.3 IBIs) were compared to the asynchronies achieved on all other trials. None of these tests yielded significant results (at $\alpha =0.02$), *Z* = 0.71, *p* = 0.48 (position), *Z* = 2.07, *p* = 0.04 (velocity), *Z* = 0.05, *p* = 0.96 (acceleration), indicating that note synchronization success did not depend on the temporal alignment of leaders’ and followers’ head gestures.

**Fig. 8:**
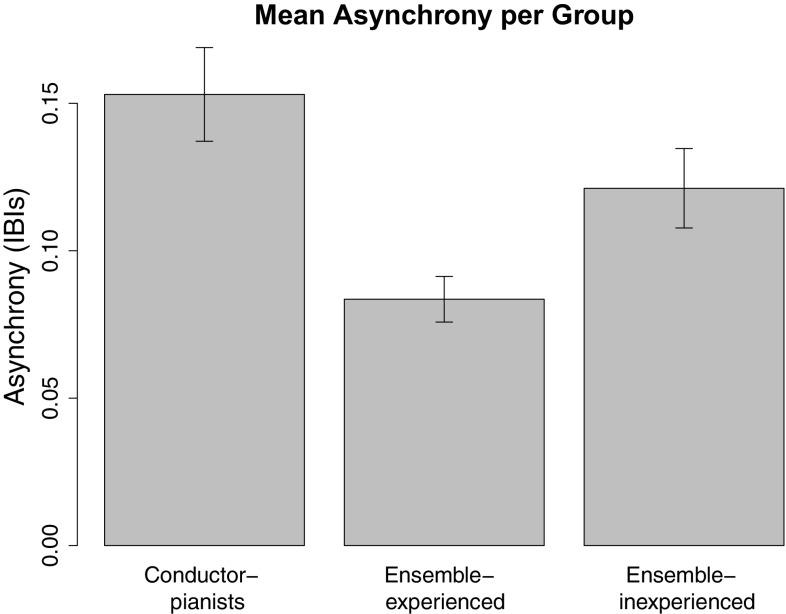
*Bars* indicate the mean asynchronies per piece achieved by conductor-pianist, ensemble-experienced, and ensemble-inexperienced duos during the interactive duo task. *Error bars* indicate standard error

#### Effects of ensemble and conducting experience on note synchrony in duo performance

Leader experience was found to affect the quality of synchronization by participants in the gesture-following task (see above). An ANOVA was run on the absolute mean asynchronies achieved by participants in the interactive duo performance task, on the first onset of each piece, to test whether the same between-group differences would emerge. It was expected that the experience shared by members of the conductor-pianist and ensemble-experienced duos would enable both better leading and better following than was the case for ensemble-inexperienced duos, resulting in more successful synchronization among conductor-pianist and ensemble-experienced groups. The effect of ensemble experience was not significant, however, *F*(1, 265) = 2.93, *p* = 0.09. Figure 8 shows the mean asynchronies achieved by duos in each group.

## General discussion

This study aimed to identify the kinematic landmarks in pianists’ cueing-in gestures that indicate beat position and the kinematic parameters that improve gesture synchronizability. Motion capture recordings were made of pianists’ upper body movements as they performed short passages under assigned leader/follower conditions. Audiovisual recordings of leaders’ performances were then presented to an independent sample of musicians, who tapped in synchrony with the beat of the music. As explained below, communicated beats occurred near points of backwards-to-forwards path reversal (head position peaks), and, with still greater reliability, near acceleration peaks. Gesture synchronizability was influenced by leader experience and gesture jerk, magnitude, and prototypicality.

### Kinematic landmarks indicating beat position

Leaders’ first onsets aligned more precisely with acceleration peaks than with other kinematic landmarks, as evidenced by the high percentage of average acceleration peak-to-onset intervals that approximated 1 IBI. There was also some alignment with position peaks, but no evidence of alignment with position troughs, suggesting that head position also makes some contribution to the communication of beats. The first onsets performed by followers during the interactive duo performance task likewise aligned most reliably with peaks in leaders’ head acceleration, with a high percentage of average acceleration peak-to-onset intervals approximating 1 IBI. Acceleration peaks preceded both leaders’ and interactive duo followers’ first onsets by slightly less than 1 IBI. Alignment with position peaks was again more reliable than alignment with position troughs, indicating that beats tend to be communicated when leaders are near points of backwards-to-forwards path reversal.

The first taps performed by followers during the gesture-following task showed greater alignment with position peaks than with position troughs, as was the case for participants in the interactive duo performance task. In contrast to recording followers, however, participants in the gesture-following task showed even more reliable alignment with acceleration troughs than with acceleration peaks (63% of average trough-to-onset intervals and 50% of average peak-to-onset intervals approximated 0 or 1 IBI). Gesture-following task participants' first taps followed acceleration peaks by slightly more than 1 IBI and acceleration troughs by slightly less than 1 IBI, suggesting that perceived beats may have fallen in between these points.

The slight delay in gesture-following task participants’ first taps relative to interactive duo followers’ first onsets, apparent in the shifted distribution medians, was presumably a result of the difference in task completed by the two groups. The reduced information available to followers during the gesture-following task meant that only movement cues could be used to predict piece onsets. The access followers in the interactive duo performance task had to other cues, such as facial expressions and the sound of breathing, might have influenced their prediction processes, leading to earlier onsets.

The significance of acceleration patterns in communicating beat position may relate to the kinematics of sound-producing gestures. The sound-producing striking gestures used in drumming or piano-playing are similar in form to the head-nodding gestures studied here, as in both cases, changes in gesture trajectory are involved in communicating beats. Research on air drumming has shown that, when people are instructed to mime drumming gestures in synchrony with a sounded rhythm, acceleration peaks in their hand gestures slightly precede audio onsets, while “hits” (points of downwards-to-upwards path reversal) lag slightly (and more variably) behind audio onsets (Dahl 2014). Sharp decelerations from peak acceleration points, therefore, indicated beat position. In piano-playing, peaks in finger acceleration correspond to moments of key impact and, as such, sounded beats (Dalla Bella & Palmer, 2011; Goebl & Palmer, 2008). If peak accelerations typically lead the sound onsets produced by percussive sound-producing gestures, then intrinsic knowledge of this association could shape our performance and perception of non-sound-producing gestures, including the cueing-in gestures intended to communicate timing information.

### Maximizing gesture synchronizability

Our finding that gesture synchronizability improved with increasing gesture smoothness and magnitude is in line with prior research suggesting that synchronization is more successful with averaged conductor gestures that are low in jerk (Wöllner et al., 2012). During joint action tasks, people tend to reduce the variability and increase the magnitude of their gestures, and the positive effects of gesture smoothness and magnitude observed here show that gesture predictability can improve as a result. These effects might have played a particularly strong role in the context of the gesture-following task, given that leaders’ upper body movements were the only source of timing information prior to piece onset. Normally, facial expressions are also involved, and likely to help with securing the follower’s attention and discriminating the cueing-in gesture from other preparatory gestures. The sound of the leader’s breathing also often acts as a cue to piece onset, but was not part of the audio presented to followers during the gesture-following task. These factors, along with the inability of followers to interact with the leader, would account for the much higher variability in synchronization success that gesture-following task participants achieved, relative to recording followers.

Contrary to our hypothesis, gesture prototypicality related to a decline, rather than an improvement in gesture synchronizability. Our measure of prototypicality (i.e., the average lag-0 cross-correlation coefficient for each gesture) gave preference to flatter, less distinctive curves. Given the relationship between gesture magnitude and synchronizability, it is therefore not surprising that synchronization was less successful with gestures scoring high in prototypicality. We should also note that none of the pianists in this study performed gestures with particularly idiosyncratic trajectories, in contrast to our previous study, in which a few musicians displayed noticeably idiosyncratic movement styles. Future studies might specifically recruit such individuals in order to disentangle the effects of gesture magnitude from individuality in movement style.

The hypothesis that synchronization would be more successful among interactive duo performance task participants when the follower’s movements mirrored the leaders’ movements was not supported. Both this and our hypothesis that synchronizability would improve with increasing gesture prototypicality derived from the idea that observers use their own motor systems to interpret and predict others’ gestures. While there was evidence that some leader–follower pairs performed head movements that were similar in form and closely aligned in time, leader–follower gesture alignment had no effect on the success of note synchronization. Different results might arise when cueing gestures are more directly tied to sound onset. For example, when pianists perform together on a single piano, they often use exaggerated wrist movements to help synchronize chords. Aligning wrist movements could help pianists regulate their timing and prove beneficial for note synchronization.

During the gesture-following task, synchronization was more successful with gestures performed by pianists who had either conducting experience or substantial experience performing in small ensembles than with gestures performed by pianists who had little ensemble experience. This difference in synchronization success suggests that conducting and ensemble performance experience improve pianists’ cueing gestures similarly. During the interactive duo performance task, slightly superior synchronization was observed among ensemble-experienced duos. The absence of similarly enhanced synchronization among conductor duos suggests that pianists whose cueing experience comes from conducting, rather than ensemble-playing, may be skilled at leading but less skilled at following. In the literature, it has been suggested that good duo coordination may depend on at least one member of the pair having good ensemble “following” skills, which include strong anticipation and timing adaptation abilities (Keller, 2008).

The reduced form of the gesture-following task presented musicians with a situation that differed substantially from normal duo performance. Followers were forced into an exclusive follower role, in which their responses had no effect on leaders’ behaviour. Normally, people attempting to synchronize their actions will adapt to each other, regardless of their assigned role (Goebl & Palmer, 2009; Konvalinka, Vuust, Roepstorff, & Frith, 2010). Followers were also forced to rely exclusively on leaders’ movement cues to predict piece onsets, while in normal performance conditions, a range of other cues would be available, including facial expressions and the sound of breathing. Pianists who participated in the interactive duo performance task were not told how their performances would be presented during the gesture-following task, so their movements were natural, and not deliberately exaggerated. Future research should investigate how much use musicians make of their co-performers’ gestures when other cues are available. It should also be noted that the performance arrangements to which this study is most relevant are those in which performers have a direct view of each other, and in the future, other viewing angles should be considered.

### Conclusions

The results of this study show that acceleration patterns communicate beats in skilled musicians’ cueing-in gestures. Musically-trained observers aligned their performed beats with the periods of sharp deceleration that followed acceleration peaks. The communicative quality of cueing gestures depended on their smoothness, magnitude, and prototypicality; both ensemble performance and conducting experience improved the quality of cueing gestures given.

These results may have implications for artificial musical systems employing gesture modelling and recognition functions. In recent years, there has been increased interest in the development of systems that people can interact with musically, some of which use gesture recognition to modulate sound output. Identification of which kinematic landmarks in musicians’ gestures indicate beat positions would benefit systems designed to synchronize discrete output with users’ rhythmic body movements (Dahl, 2014). Some recently-developed systems, designed to fill the role of an accompanist or duet partner, output music in real time to accompany human performances. Output timing is continuously adjusted in response to fluctuations in the human performer’s timing to maintain coordination. There have been some attempts to introduce expressive and receptive visual communication capabilities to such systems in the form of prescribed gestures, given at prescribed times (Maezawa & Yamamoto, 2016), but an improved understanding of how beats are communicated naturally and how to communicate beats clearly would allow for more robust and natural communication between the system and the human performer.

## Footnotes

1 We had previously measured the average audio-visual asynchrony in stimuli presented on this computer, in Presentation, to be 3 ms (SD = 10), as described in Bishop and Goebl (2015). Images had been imported into VideoMach at 120 fps, so temporal offsets between audio and video track durations were never more than 8 ms (half the sampling period of the displayed videos).

2 To confirm that gesture-following participants used the videos to predict piece onsets and did not merely respond to audio onsets, the leader–follower first beat asynchronies achieved during familiarization blocks were calculated and compared to the leader–follower first beat asynchronies achieved during experimental blocks. First taps lagged further behind leader onsets during familiarization blocks than during experimental blocks, *Z* = 4.20, *p* < 0.001, *r* = 0.12 [familiarization block asynchrony magnitude *M* = 0.51 (SD = 0.97), experimental block *M* = 0.12 (SD = 0.80)], indicating that the videos were useful in predicting piece onsets.
